# The effectiveness of additional thoracic paravertebral block in improving the anesthetic effects of regional anesthesia for proximal humeral fracture surgery in elderly patients: study protocol for a randomized controlled trial

**DOI:** 10.1186/s13063-020-4078-9

**Published:** 2020-02-19

**Authors:** Xiaofeng Wang, Hui Zhang, Zhenwei Xie, Qingfu Zhang, Wei Jiang, Junfeng Zhang

**Affiliations:** Department of Anesthesiology, Shanghai Jiao Tong University Affiliated Sixth People’s Hospital, 600 Yishan Road, Shanghai, 200233 China

**Keywords:** Brachial plexus block, Cervical plexus block, Elderly, Intercostobrachial nerve, Proximal humeral fracture, Regional anesthesia, Thoracic paravertebral block

## Abstract

**Background:**

The innervation of the shoulder-upper-extremity area is complicated and unclear. Regional anesthesia with a brachial plexus and cervical plexus block is probably inadequate for the proximal humeral surgery. Missing blockade of the T1–T2 nerves may be the reason. We conduct this prospective randomized controlled trial (RCT) to explore whether an additional T2 thoracic paravertebral block (TPVB) can improve the success rate of regional anesthesia for elderly patients in proximal humeral fracture surgery.

**Methods/design:**

The patients aged 65 years or older, referred for anterior-approach proximal humeral fracture surgery, will be enrolled. Each patient will be randomly assigned 1:1 to receive a combined interscalene brachial plexus with superficial cervical plexus block (IC) (combined interscalene brachial plexus with superficial cervical plexus block) or an IC block combined with thoracic paravertebral block (ICTP) block (combined thoracic paravertebral block with brachial plexus and superficial cervical plexus block). The primary outcome is the success rate of regional anesthesia without rescue analgesic methods. The secondary outcomes are as follows: sensory block at the surgical area, proportion of patients who need rescue anesthesia (intravenously administered remifentanil or conversion to general anesthesia), cumulative doses of intraoperative vasoactive medications and adverse events. The total sample size is estimated to be 80 patients.

**Discussion:**

This RCT aims to confirm whether an additional T2 TPVB can provide better anesthetic effects of regional anesthesia with brachial and cervical plexus block in elderly patients undergoing proximal humeral surgery.

**Trial registration:**

ClinicalTrials.gov, ID: NCT03919422. Registered on 19 April 2019.

## Background

Proximal humeral fractures account for 4–10% of all fractures occurring in the elderly population over 60 years, with the greatest incidence in women aged 80 to 89 years [1, 2]. These fractures possibly affect quality of life and are related to high mortality [3]. The aged patients are commonly afflicted with severe cardiac or pulmonary co-morbidity, which may increase their perioperative risks. For the high-risk patients who require surgical treatments, the choice of anesthesia is a challenge. Compared with general anesthesia (GA), regional anesthesia can provide more stable hemodynamics and effective opioid-free analgesia [4]. It is also associated with a relatively lower incidence of perioperative complications, shorter postoperative stays and greater patient satisfaction [5–8].

The understanding of anatomy and innervation in surgical area is the prerequisite for a well-performed nerve block. The shoulder joint is predominantly innervated by the suprascapular nerve, axillary nerve (C5–6) and lateral pectoral nerves (C7). Part of the anterior surface of the shoulder is innervated by the supraclavicular nerve (C3–4). Therefore, blockade of the brachial plexus and the cervical plexus (IC block) is basically required [9]. But the innervation of the shoulder-proximal upper extremity area is not exactly the same as that of shoulder joint. This is an area where the cervical, brachial and thoracic nerves meet together and the nerve distribution requires extensive local anesthetic coverage [10, 11]. An IC block might not cover the comprehensive dermatome distribution to provide adequate anesthesia for every patient undergoing proximal humeral fracture surgery. Our pilot study found that 40% of patients who received an IC block complained of pain and needed intravenously administered (IV) narcotics or local infiltration, even conversion to general anesthesia (unpublished data). We know that an interscalene brachial plexus block (ISPB) cannot anesthetize the medial part of upper extremity, which is innervated by the T1–T2 segments. T1–T2 thoracic nerves commonly contribute to the brachial plexus, but there is no identical innervation pattern at the shoulders of all the patients due to the anatomical variation [12]. Therefore, they may co-innervate the surgical area with the brachial and cervical plexus in a portion of population. Missing blockade of T1–T2 probably leads to inadequate anesthesia in some patients after simply combined brachial with cervical plexus block.

In the peripheral branches of the T1–T2 segments, the intercostobrachial nerve (ICBN) most possibly involves the innervation of this surgical area. It is responsible for the sense of the upper half of the anteromedial area of the upper extremity. The ICBN mainly originates from T2 with an occasional contribution from T1 and T3. Some peripheral techniques to block ICBN, such as ultrasound-guided selective block [13, 14], pectoral nerve block (PECS II) [15–17] and subcutaneous ring infiltration [18], have been described in the literature. However, the efficacy of these techniques are not certain owing to the variations of the ICBN at the axilla [19, 20]. Except for the ICBN, whether other branches of T1–T2 involving the innervation are unclear, the T1–T2 segments require additional blocking because the usual approaches for brachial plexus anesthesia cannot block them. The thoracic paravertebral block (TPVB) is a regional anesthesia technique that can be used in thoracic, cardiac or breast surgery and its effectiveness has been demonstrated in many studies [21–25]. Even so, whether adding T2 TVPB on the basis of the IC block can provide more definite anesthetic effects at the shoulder-upper-extremity area has not been sufficiently investigated. Therefore, this study is designed to assess the effectiveness of additional T2 TPVB in improving the success rate of regional anesthesia in elderly patients undergoing anterior-approach proximal humeral surgery.

## Methods/design

### Trial design and setting

**Fig. 1:**
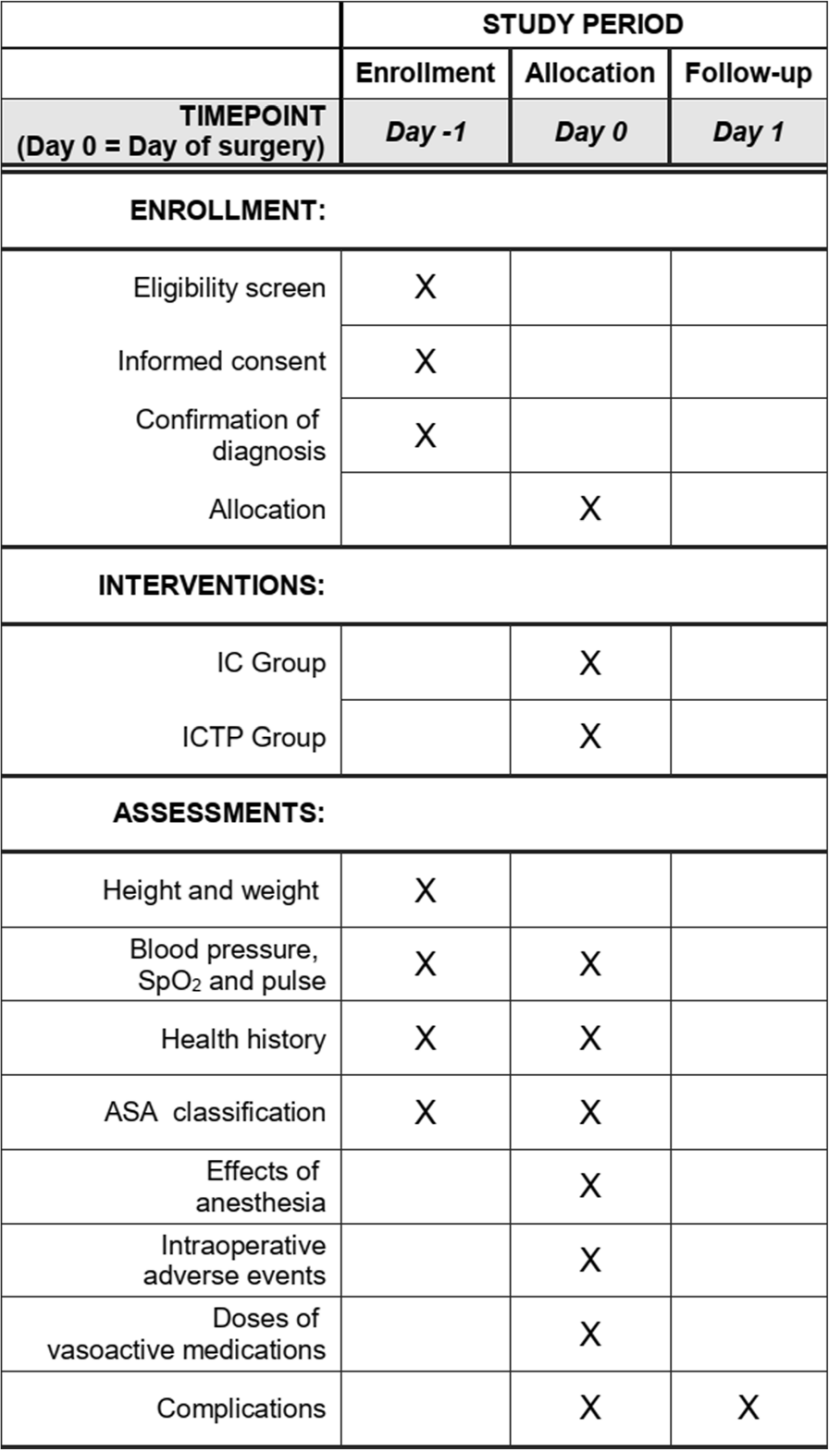
Standard Protocol Items: Recommendations for Interventional Trials (SPIRIT) recommended content for the schedule of enrollment, interventions and assessments

**Fig. 2:**
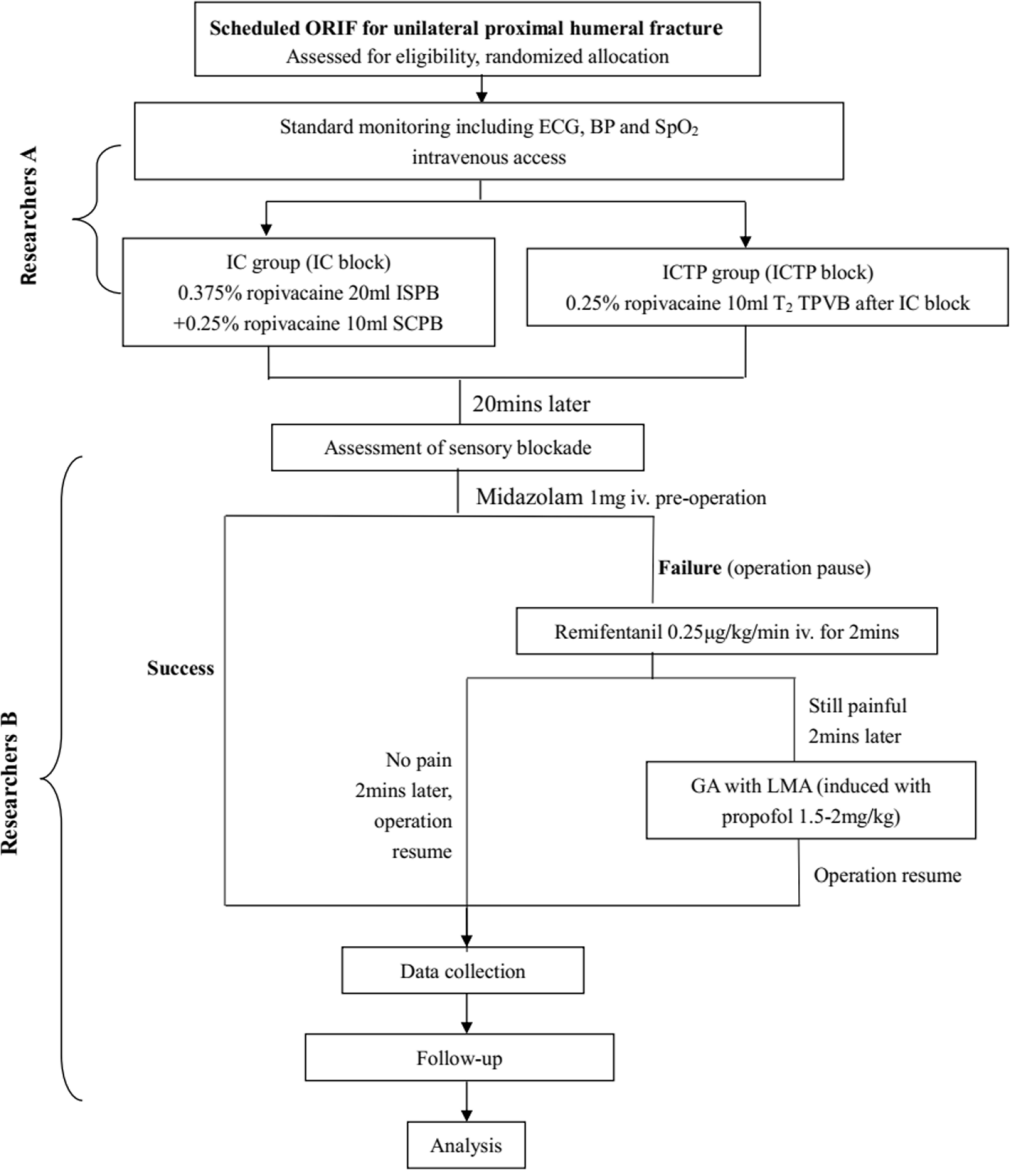
Flowchart of trial procedures

This prospective, two-armed, parallel RCT will be performed at Shanghai Jiao Tong University Affiliated Sixth People’s Hospital, China. The study is developed based on the Standard Protocol Items: Recommendations for Interventional Trials (SPIRIT) 2013 Statements, Fig. 1 (the SPIRIT Checklist is available as Additional file 1) [26]. The Consolidated Standards of Reporting Trials (CONSORT) flow diagram will be followed in reporting the final results of this trial. A flowchart of the trial design is shown in Fig. 2.

### Informed consent

Written informed consent will be obtained from each patient before enrollment. They will be informed that they are free to withdraw their consent from the study at any time. The procedure, benefits, risks, and data management of this study will be clarified in detail for the participants during the preoperative conversation.

### Participants and recruitment

Elderly patients scheduled for open reduction and internal fixation (ORIF) for a unilateral proximal humeral fracture will be recruited and screened for eligibility. An independent researcher (QZ) will finish the recruitment when performing the preoperative interview 1 day before the surgery.

Inclusion criteria:

- Written informed consent is obtained from the patient or patient’s legal representative
- Age ≥ 65 years old
- Body mass index (BMI) < 30 kg/m^2^
- American Society of Anesthesiologists (ASA) classification I–II
- Anterior approach of the operative incision

Exclusion criteria:

- Request for general anesthesia
- Nerve block is unable to be performed due to various reasons (open trauma, hematoma or skin infection at the blocking area)
- Coagulation dysfunction or anticoagulation therapy
- History of upper-limb nerve injury or phrenic-nerve injury
- Multiple trauma
- Uncontrolled respiratory disease (severe chronic obstructive pulmonary disease, asthma, pulmonary infection, pneumothorax, etc.)
- Uncontrolled hypertension (systolic pressure over 180 mmHg or diastolic pressure over 110 mmHg)
- Uncontrolled heart disease (moderate and severe coronary heart disease, valvular disease or arrhythmia, etc.)
- Stroke or cognitive dysfunction (unable to communicate or cooperate)
- Hypersensitivity or allergy to anesthetics (ropivacaine or remifentanil)

### Randomization and blinding

Random allocation will be performed by a researcher (HZ) before the trial using a randomization sequence (generated on http://www.randomization.com). The allocation concealment strategy is achieved with sequentially numbered, opaque, sealed envelopes until confirming the inclusion/exclusion criteria. After the envelopes have been opened sequentially, the patients will be randomly assigned in a 1:1 ratio to receive an IC block (combined interscalene brachial plexus with superficial cervical plexus block) or an ICTP block (combined thoracic paravertebral block with brachial plexus and cervical plexus block). The envelopes will be resealed after confirming the allocation. As the nerve block intervention cannot be blinded from patients and staff implementing the intervention, only the outcome assessor (ZX) will be kept blinded to the randomized allocation and intervention. He will not be present in the operation theatre until the nerve block is finished. Emergency un-blinding rules will be applied for the outcome assessor if a serious adverse event (total spinal block or pneumothorax) occurs during the surgery.

### Interventions

All the patients will undergo preoperative fasting for 8 h and water deprivation for 2 h. After placement of standard ASA monitors, intravenous access for fluid infusion will be established in the forearm. No sedatives or IV narcotics will be given prior to the block. The patient will receive an ultrasound-guided IC block or an ICTP block according to the allocation. The block will be performed following standard skin disinfection with a SonoSite S-Nerve™ ultrasound machine (Bothell, WA, USA). Local lidocaine (1%) for skin numbing will be given prior to insertion of the block needle. The entire nerve-block procedure of all the patients will be performed by the same anesthesiologist, who is skilled in performing ultrasound-guided regional anesthesia (XW).

In the IC group, combined interscalene brachial plexus and superficial plexus block will be performed as follows.

The patient will be placed in the lateral decubitus position with the operative side upwards. A linear array transducer (6–13 MHz) with a sterile cover and a 22-gauge (G) block needle (KDL™, Kindly group, Shanghai, China) will be used. An in-plane approach, advancing the needle along the longitudinal axis of the transducer and visualizing the entire shaft, will be employed. Twenty milliliters (ml) of 0.375% ropivacaine (Naropin™, AstraZeneca AB, Gothenburg, Sweden) will be injected between the superior and middle trunk of the brachial plexus at the C7 level to reduce the likelihood phrenic-nerve palsy. The transducer will be then moved cephalad until the superficial cervical plexus emerges from the C4 intervertebral foramen. Ten milliliters of 0.25% ropivacaine will be injected to block the nerve [27–29]. The total dose of ropivacaine for IC group will be 100 mg.

In the ICTP group, the procedure will be performed as follows.

**Fig. 3:**
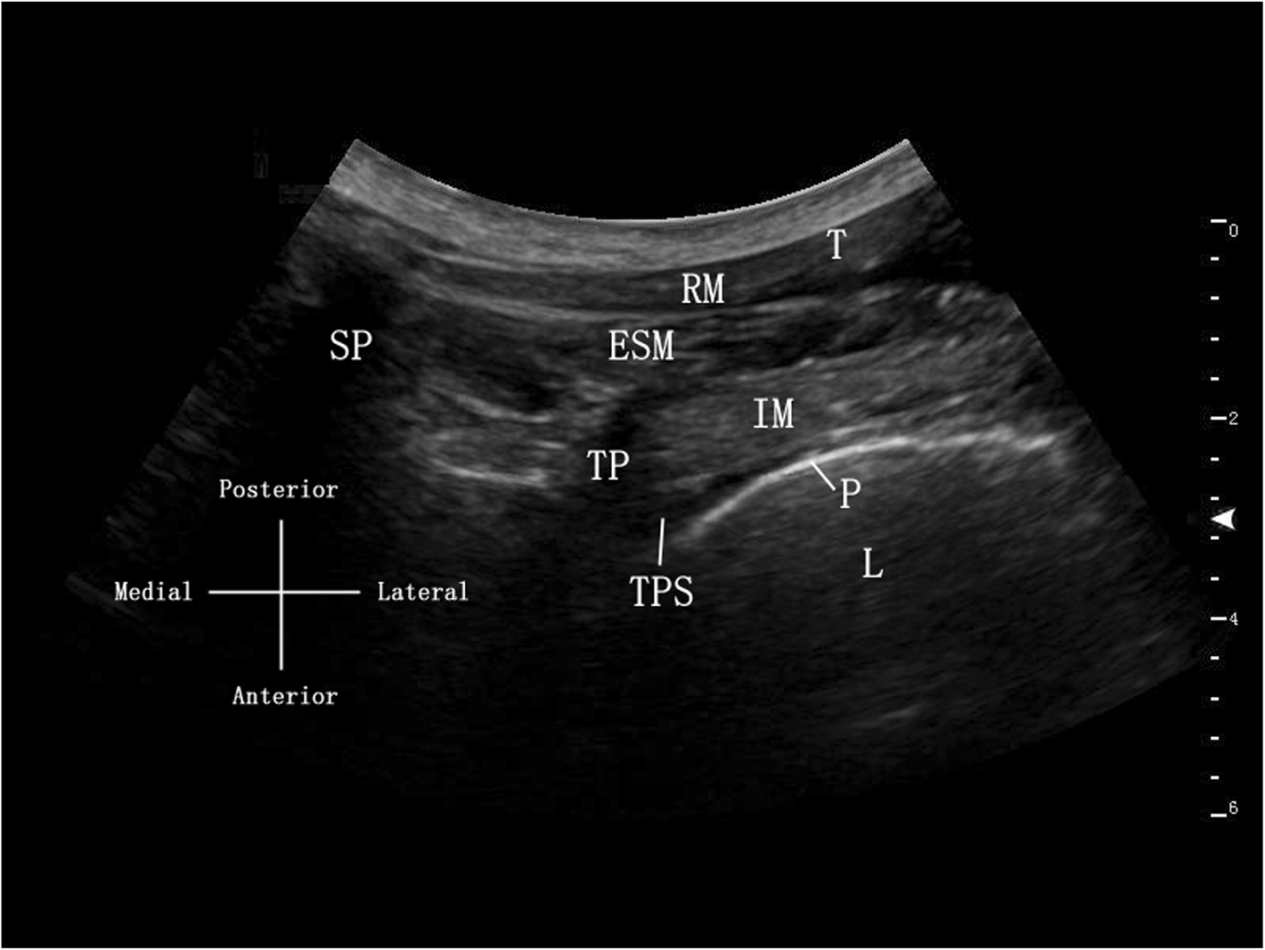
Ultrasound image of the thoracic paravertebral block (TPVB). *TPS* thoracic paravertebral space, *TP* transverse process, *SP* spinous process, *P* pleura, *L* lung, *IM* intercostal muscle, *RM* rhomboid muscle, *ESM* erector spinae muscles, *T* trapezius

On the basis of the combined brachial plexus and superficial plexus block, an additional T2 TPVB will then be performed. The T_2–3_ intervertebral space should be determined by ultrasound-image scanning and palpation counting from the C7 spinous process. A curve array transducer (2–5 MHz) will be placed at the T_2–3_ intercostal level with a slightly oblique scan to visualize the transverse process, costotransverse ligament, internal intercostal membrane and parietal pleura (Fig. 3). A 10-cm, 22-G needle will be introduced into the thoracic paravertebral space beyond the internal intercostal membrane with its tip positioned outside the transverse process. Following negative aspiration of air, blood or cerebrospinal fluid in the needle, 10 ml 0.25% ropivacaine will be injected into the paravertebral space [30, 31]. The total dose for ICTP group will be 125 mg.

Then the patient will be placed in the supine position. Twenty minutes later, after the sensory block at the surgical area is assessed, the patient will be transferred to the operating room and placed in a beach-chair position. One milligram of midazolam will be given IV. Oxygen will be routinely given via a nasal catheter at a flow rate of 3 L/min until the end of operation. Remifentanil (50 μg/ml), propofol (10 mg/ml) and a laryngeal mask airway (LMA) will be prepared and given when there is inadequate analgesia. The anesthetic effects will be assessed since the operation began: (1) if it is successful, the operation will be continued; (2) if it is inadequate, the operation will be paused and remifentanil will be given IV at a rate of 0.25 μg/kg/min. Two minutes later, the operation will be continued if adequate anesthetic effects are achieved. The rate of IV remifentanil can be appropriately regulated (no more than 0.25 μg/kg/min) in the following operation according to the end-tidal carbon dioxide pressure (P_ET_CO_2_) and respiratory rate of the patient. On the contrary, the inadequately anesthetized patient will be induced with propofol (1.5–2 mg/kg) for converting to GA with LMA. Inhaled sevoflurane will be used to maintain anesthesia during the operation. The patient who receives a GA will be transferred to the post-anesthesia care unit (PACU) after the operation. If no GA is required, the patient will be sent to the ward.

### Intraoperative monitoring and management

Blood pressure, heart rate and pulse oxygen saturation (SpO_2_) will be recorded throughout the operation. Respiratory rate and P_ET_CO_2_ will be monitored via an intranasal catheter connected to the monitor. Intraoperative mean arterial pressure (MAP) higher (or lower) than 30% from the baseline value will be defined as hypertension (or hypotension). Hypotension will be treated promptly with IV ephedrine 5–10 mg or deoxyepinephrine 50–100 μg, while hypertension will be treated with urapidil 5–10 mg. Bradycardia (defined as a heart rate < 60 beats/min) will be treated with IV atropine 0.5 mg. Other adverse events including dyspnea and pneumothorax will also be recorded. Dyspnea caused by phrenic-nerve palsy or remifentanil infusion will be supported with mask ventilation or a reducing dose of remifentanil. The absolute risk of pneumothorax under ultrasound-guided TPVB is low and it has never happened before in our center. However, it is one of the most serious potential complication caused by TPVB and the patients must be screened with clinical monitoring. Chest fluoroscopy and ultrasound will be used to eliminate pneumothorax if aggravated hypoxemia happens. Closed thoracic drainage then may be administered according to the severity of pneumothorax.

### Outcome definitions

#### Primary outcome evaluation

The primary outcome is the success rate of regional anesthesia with pain-free surgery, which will be recorded as “successful” and “inadequate.” “Successful” is defined as the ability to finish the operation without rescue anesthesia (IV narcotics, general anesthesia or local infiltration by the surgeon, etc). The patient who complains of pain during the operation will be defined as having “inadequate” pain control.

#### Secondary outcome evaluations

- Assessment of sensory block at surgical area. (This will be evaluated on a 3-point rating scale (0 = normal sensation, 1 = decreased sensation and 2 = no perception) 20 min after nerve block, using a pinprick and an alcohol swab, respectively. The testing area will be divided into four portions: distal clavicle area, deltoid area, upper medial and upper lateral area of the upper extremity)
- Proportion of patients completing the surgery with remifentanil
- Proportion of patients converting to GA with LMA
- Cumulative doses of intraoperative vasoactive medications (urapidil, atropine, ephedrine and deoxyepinephrine, etc.)
- Complications related to anesthesia (local anesthetic systemic toxicity, pneumothorax, epidural block, total spinal block, hematoma, etc.)
- Intraoperative adverse reactions (hypertension, hypotension, bradycardia, tachycardia, dyspnea, etc.)

### Participant timeline

For a given participant, enrollment will be performed 1 day prior to surgery and confirmed again on the day of surgery. Then random allocation will be assigned by HZ before intervention. The participant will be followed up for postoperative complications on 1 day after surgery. The accrual period of this trial is expected to be about 1 year. The timeline is shown in Fig. 1.

### Sample size calculation

Calculation of the sample size is based on the primary outcome. A study including 27 patients who underwent shoulder or upper-extremity surgery using brachial plexus block showed that the success rate was 85.2% [32]. In our study, only patients who undergo anterior-approach ORIF for proximal humeral fracture will be included. So we assume that the actual success rate of the IC group will be lower than that in the previous study. On the other hand, we conducted a pilot study with 10 patients in each group. The success rate achieved was 60% in the IC group and 90% in the ICTP group (unpublished data). Therefore, using the formula of Two Independent Sample Rates (Testing Two Proportions using the *Z*-Test with Pooled Variance), a sample size of 32 for each group will achieve 80% power to detect the difference with a two-tailed 5% significance level. Then the total sample size will be 80 including the possible missing (20%).

### Statistical analysis

Statistical data analyses will be performed on an intention-to-treat basis, including all participants as randomized, except whose who withdraw consent for the use of their data [33]. Numerical variables, such as patient characteristics and surgery data, will be expressed as mean ± standard deviation (SD) or median (interquartile range). The normally distributed numerical data will be compared using Student’s unpaired *t* test, whereas non-parametric data will be compared using the Mann-Whitney *U* test. Categorical variables, such as success rate and sensory block at surgical area, will be expressed as frequency (%). A chi-square test or Fisher’s exact test will be used for categorical variables. The statistical analysis will be performed with SPSS V.24.0 (IBM Corporation, Armonk, New York, USA). A two-tailed, *P* < 0.05 will be considered statistically significant.

### Data collection, monitoring and management

Preoperative, intraoperative and 1-day postoperative follow-up data will be collected from electronic medical records, monitoring machines and relevant manual records by the research staff (ZX). All electronic and handwriting data will be stored on a password-protected computer. Data and safety monitoring will be the responsibility of the principle investigator (XW) and study director (JZ).

### Harms

All the severe adverse events related to the study intervention will be recorded in the study database and reported as required to Shanghai Jiao Tong University Affiliated Sixth People’s Hospital Institutional Review Board.

### Auditing

No formal auditing process is proposed for this trial.

### Participant retention and withdrawal

All reasonable efforts will be made to ensure optimum participant engagement and to reduce study attrition. However, the study involves an intention-to-treat analysis. Therefore, all participants will have the right to withdraw from the study at any stage. If the participant is willing to provide them, any data already collected from that participant will be analyzed.

### Data retention

To enable evaluations and audits from regulatory authorities, data obtained from participants will retained confidential and stored securely at the Department of Anesthesiology of Shanghai Jiao Tong University Affiliated Sixth People’s Hospital for a minimum of 5 years. The investigators will keep records including the identity of all participants, all original signed informed consents, serious adverse event recordings and case report forms. The data will be kept safely and not revealed to other people without appropriate permission.

### Protocol amendments

Any change in the study protocol will require an amendment. Any proposed protocol amendments will be initiated by the principal investigators. All amended versions of the protocol will be signed by the staff in the study and the amendment forms will be submitted to the Ethics Committee for approval.

### Trial dissemination

The outcomes of the study will be disseminated in a peer-reviewed journal or at scientific conferences.

## Discussion

Ultrasound-guided brachial plexus and cervical plexus block is probably inadequate for the anesthesia of proximal humeral-fracture surgery. T2 TPVB is performed near the ventral root of the second thoracic nerve and the anesthetic solution can spread to T1 and T3 along the limited thoracic paravertebral space. We intend to block the branches of the T1–T2 segments by T2 TPVB. In this trial, our primary purpose is to evaluate the anesthetic effects of additional T2 TPVB in the elderly patients undergoing proximal humeral surgery. The anesthetic effects will be mainly assessed by the success rate of regional anesthesia, which is the most convincing direct evidence. Meanwhile, the sensory block at the surgical area will be indirect evidence to evaluate the anesthetic effects. The upper medial area of the upper extremity will be tested in order to confirm the anesthetic effect of the T2 TPVB technique. The purpose of sensory assessment in the other three areas is to eliminate the influence on primary outcome evaluation from inadequate blockade of the brachial or cervical plexus. These areas are innervated by the suprascapular nerve, axillary nerve and supraclavicular nerve. Combined sensory assessment of the dermatome with actual anesthetic success rate will be helpful for us to better understand the contribution of the T1–T2 nerves for proximal humeral surgery. However, there may be a bias in assessing the primary outcome because the patient will know the treatment that they receive. To reduce the influence, the outcome assessor will be kept blinded throughout the operation.

AS well as the benefits, the potential risks of TPVB performed in elderly patients should also be taken into consideration. As the paravertebral space is close to the pleura, an important issue concerning the TPVB is obviously a reasonable degree of safety regarding pleural puncture and pneumothorax [34]. Also, medially, the space communicates with the epidural space via the intervertebral foramen [35]. The incidence of epidural block and total spinal block must also be recorded. In our study, the in-plane technique of the TPVB will be performed by an experienced anesthesiologist, who is skilled in ultrasound-guided regional anesthesia, to minimize the aforementioned risks. In addition to the skill and monitoring, low-concentration ropivacaine will be used to reduce the toxicity. Nevertheless, the safety and necessity of additional TPVB in the elderly patients must be carefully assessed by analyzing the risks and benefits. The proportion of patients who need rescue anesthesia is a useful reference to evaluate the necessity of TPVB. We will observe whether the patients with inadequate anesthetic effects can be rescued by a low dose of opioids. The results can help us to determine the indispensability of this potentially risky technique.

In conclusion, this trial should enable us to better assess the effectiveness of regional anesthesia in the elderly population undergoing proximal humeral fracture, with the potential possibility of avoiding opioids or general anesthesia. It may provide us an with ideal combination of nerve blocks for the surgery at the boundary of the shoulder-upper-extremity area. It should also advance the understanding of innervation in this surgical area.

### Trial status

At the time of manuscript submission, the study had been launched and a few patients had participated in the trial. The current version of protocol was 1.1 on 21 March 2019. The recruitment was began on 5 May 2019 and is expected to be completed in April 2020.

## Supplementary information

**Additional file 1..** SPIRIT 2013 Checklist: Recommended items to address in a clinical trial protocol and related documents*

## Data Availability

All investigators will have access to the final de-identified study dataset from the corresponding author for the purpose of scientific publications.

## Abbreviations

ASA: American Society of Anesthesiologists
BMI: Body mass index
CONSORT: Consolidated Standards of Reporting Trials
G: Gauge
GA: General anesthesia
IC: Combined interscalene brachial plexus with superficial cervical plexus block
ICTP: IC block combined with thoracic paravertebral block
ISPB: Interscalene brachial plexus
IV: Intravenously administered
LMA: Laryngeal mask airway
MAP: Mean arterial pressure
ORIF: Open reduction and internal fixation
PACU: Post-anesthesia care unit
SCPB: Superficial cervical plexus block
SPIRIT: Standard Protocol Items: Recommendations for Interventional Trials
SpO_2_: Pulse oxygen saturation
TPVB: Thoracic paravertebral block

## References

[1] Bell J-E, Leung BC, Spratt KF, Koval KJ, Weinstein JD, Goodman DC, Tosteson ANA (2011). Trends and variation in incidence, surgical treatment, and repeat surgery of proximal humeral fractures in the elderly. J Bone Joint Surg Am.

[2] Bahrs C, Bauer M, Blumenstock G, Eingartner C, Bahrs SD, Tepass A (2013). The complexity of proximal humeral fractures is age and gender specific. J Orthop Sci.

[3] Slobogean GP, Johal H, Lefaivre KA, MacIntyre NJ, Sprague S, Scott T (2015). A scoping review of the proximal humerus fracture literature. BMC Musculoskelet Disord.

[4] Mirza F, Brown AR (2011). Ultrasound-guided regional anesthesia for procedures of the upper extremity. Anesthesiol Res Pract.

[5] Hausman MS, Jewell ES, Engoren M (2015). Regional versus general anesthesia in surgical patients with chronic obstructive pulmonary disease: does avoiding general anesthesia reduce the risk of postoperative complications?. Anesth Analg.

[6] Brox WT, Chan PH, Cafri G, Inacio MCS (2016). Similar mortality with general or regional anesthesia in elderly hip fracture patients. Acta Orthop.

[7] Xu R, Lian Y, Li WX (2016). Airway complications during and after general anesthesia: a comparison, systematic review and meta-analysis of using flexible laryngeal mask airways and endotracheal tubes. PLoS ONE.

[8] Herrick MD, Liu H, Davis M, Bell J-E, Sites BD (2018). Regional anesthesia decreases complications and resource utilization in shoulder arthroplasty patients. Acta Anaesthesiol Scand.

[9] Conroy PH, Awad IT (2011). Ultrasound-guided blocks for shoulder surgery. Curr Opin Anaesthesiol.

[10] Mirza Farheen, Brown Anthony R. (2011). Ultrasound-Guided Regional Anesthesia for Procedures of the Upper Extremity. Anesthesiology Research and Practice.

[11] Fredrickson MJ, Krishnan S, Chen CY (2010). Postoperative analgesia for shoulder surgery: a critical appraisal and review of current techniques. Anaesthesia..

[12] Loukas M, El-Zammar D, Tubbs RS, Apaydin N, Louis RG, Wartman C, Shoja MM (2010). A review of the T2 segment of the brachial plexus. Singap Med J.

[13] Chang Ke-Vin, Mezian Kamal, Naňka Ondřej, Wu Wei-Ting, Lou Yueh-Ming, Wang Jia-Chi, Martinoli Carlo, Özçakar Levent (2018). Ultrasound Imaging for the Cutaneous Nerves of the Extremities and Relevant Entrapment Syndromes: From Anatomy to Clinical Implications. Journal of Clinical Medicine.

[14] Magazzeni P, Jochum D, Iohom G, Mekler G, Albuisson E, Bouaziz H (2018). Ultrasound-guided selective versus conventional block of the medial brachial cutaneous and the intercostobrachial nerves: a randomized clinical trial. Reg Anesth Pain Med.

[15] Schuitemaker R JB, Sala-Blanch X, Rodriguez-Pérez CL, Mayoral R JT, López-Pantaleon LA, Sánchez-Cohen AP (2018). The PECS II block as a major analgesic component for clavicle operations: a description of 7 case reports. Rev Esp Anestesiol Reanim.

[16] Blanco R, Fajardo M, Parras Maldonado T (2012). Ultrasound description of Pecs II (modified Pecs I): a novel approach to breast surgery. Rev Esp Anestesiol Reanim.

[17] Quek KH, Low EY, Tan YR, Ong ASC, Tang TY, Kam JW, Kiew ASC (2018). Adding a PECS II block for proximal arm arteriovenous access—a randomised study. Acta Anaesthesiol Scand.

[18] Roussel J, Thirkannad S (2014). Comparison of 3 ultrasound-guided brachial plexus block approaches for cubital tunnel release surgery in 120 ambulatory patients. AANA J.

[19] Soares EWS (2014). Anatomical variations of the axilla. Springerplus..

[20] Deshmukh VR, Bhardwaj H, Khan F, Jacob TG (2017). Aberrant cutaneous nerve loops in the axilla. Acta Med.

[21] D'Ercole F, Arora H, Kumar PA (2018). Paravertebral block for thoracic surgery. J Cardiothorac Vasc Anesth.

[22] Abdallah FW, Morgan PJ, Cil T, McNaught A, Escallon JM, Semple JL (2014). Ultrasound-guided multilevel paravertebral blocks and total intravenous anesthesia improve the quality of recovery after ambulatory breast tumor resection. Anesthesiology..

[23] Head LK, Lui A, Boyd KU (2018). Efficacy and safety of bilateral thoracic paravertebral blocks in outpatient breast surgery. Breast J.

[24] Krediet AC, Moayeri N, van Geffen G-J, Bruhn J, Renes S, Bigeleisen PE, Groen GJ (2015). Different approaches to ultrasound-guided thoracic paravertebral block: an illustrated review. Anesthesiology..

[25] Koyyalamudi VB, Elliott C, Gibbs CP, Boezaart AP (2010). Perioperative analgesia for forequarter amputation in a child: a dual paravertebral approach. Anesth Analg.

[26] Chan A-W, Tetzlaff JM, Gøtzsche PC, Altman DG, Mann H, Berlin JA (2013). SPIRIT 2013 explanation and elaboration: guidance for protocols of clinical trials. BMJ..

[27] Soeding P, Eizenberg N (2009). Review article: anatomical considerations for ultrasound guidance for regional anesthesia of the neck and upper limb. Can J Anaesth.

[28] El-Boghdadly K, Chin KJ, Chan VWS (2017). Phrenic nerve palsy and regional anesthesia for shoulder surgery: anatomical, physiologic, and clinical considerations. Anesthesiology..

[29] Senapathi TGA, Widnyana IMG, Aribawa IGNM, Wiryana M, Sinardja IK, Nada IKW (2017). Ultrasound-guided bilateral superficial cervical plexus block is more effective than landmark technique for reducing pain from thyroidectomy. J Pain Res.

[30] Boezaart AP, Lucas SD, Elliott CE (2009). Paravertebral block: cervical, thoracic, lumbar, and sacral. Curr Opin Anaesthesiol.

[31] Taketa Y, Irisawa Y, Fujitani T (2018). Comparison of analgesic efficacy between two approaches of paravertebral block for thoracotomy: a randomised trial. Acta Anaesthesiol Scand.

[32] Nguyen HC, Fath E, Wirtz S, Bey T (2007). Transscalene brachial plexus block: a new posterolateral approach for brachial plexus block. Anesth Analg.

[33] Fergusson D, Aaron SD, Guyatt G, Hébert P (2002). Post-randomisation exclusions: the intention to treat principle and excluding patients from analysis. BMJ..

[34] Naja MZ, Gustafsson AC, Ziade MF, El Rajab M, Al-Tannir M, Daher M, Lönnqvist PA (2005). Distance between the skin and the thoracic paravertebral space. Anaesthesia..

[35] Luyet C, Eichenberger U, Greif R, Vogt A, Szücs Farkas Z, Moriggl B (2009). Ultrasound-guided paravertebral puncture and placement of catheters in human cadavers: an imaging study. Br J Anaesth.

